# Road Traffic Anomaly Detection by Human-Attention-Assisted Text–Vision Learning

**DOI:** 10.3390/s26092638

**Published:** 2026-04-24

**Authors:** Yachuang Chai, Wushouer Silamu

**Affiliations:** 1 School of Computer Science and Technology, Xinjiang University, Urumqi 830046, China; 2 Xinjiang Key Laboratory of Multilingual Information Technology, Urumqi 830046, China

**Keywords:** traffic anomaly detection, multi-modal learning, traffic surveillance video, video anomaly detection

## Abstract

With the rapid development of society, the number of road vehicles has increased significantly, leading to a growing severity of traffic accident issues. Timely and accurate detection of road traffic anomalies or accidents is crucial for reducing fatalities and alleviating traffic congestion. Consequently, the detection of road traffic anomalies has become a focal point of research in recent years. With the assistance of computer technologies such as deep learning, researchers have developed more accurate and effective methods for detecting road traffic anomalies. However, the small proportion of anomaly-prone areas in surveillance video frames, combined with the complex and difficult-to-capture patterns of accidents, presents new challenges for the application of deep models to traffic anomaly detection from a surveillance perspective. In light of this, this paper annotates the TADS dataset we previously proposed, a popular text-assisted video representation learning method, to develop a more efficient detection method. Utilizing the well-known video-text model CLIP, we have constructed a detection model that leverages unique text and eye-gaze annotation data from the TADS dataset to learn anomaly representations more effectively, thereby improving the detection of road traffic anomalies from a surveillance perspective. Experimental results demonstrate the superiority of our model for detecting traffic anomalies from a surveillance perspective, as well as the utility of the text and eye-gaze data included in the dataset.

## 1. Introduction

With the continuous development of society, the number of road vehicles has increased steadily, leading to a significant rise in road traffic accidents. These accidents have become a major concern for societal development and pose serious threats to human life. In recent years, numerous experts and scholars have advanced research on the detection of road traffic accidents. However, influenced by the growing field of intelligent driving, most studies have primarily focused on the driver’s perspective [1,2,3]. Although the use of road monitoring equipment has become more prevalent and intelligent computing technologies have made significant breakthroughs, research on accident detection from a surveillance perspective remains relatively scarce compared to that from a driver’s perspective. Timely detection of traffic accidents across a wide range of surveillance perspectives can facilitate immediate response measures, such as blocking affected roads and dispatching medical and rescue teams. These responses are crucial for alleviating traffic congestion and reducing casualties. Therefore, advancing research on road traffic accident detection from surveillance perspectives is of great significance. However, it is not appropriate to directly apply the detection method from the driving perspective to the surveillance perspective, because there are notable differences in the visual information under the two perspectives. For example, traffic scenes from surveillance perspectives typically show a fixed background, while driving perspectives are characterized by constant changes in the central vehicle’s surroundings. Additionally, the area of a traffic accident from a surveillance perspective usually occupies a smaller spatial region within the entire camera frame, whereas accidents from a driving perspective often cover a larger area and may exhibit significant fluctuations or even shaking, particularly in accidents involving the ego-car. Consequently, to promote research on accident detection from the surveillance perspectives, we proposed a large-scale traffic accident dataset, TADS, in our article [4]. This dataset not only consists of a substantial volume and a diverse range of accident types but also eye-gaze data that is equivalent in size to the video frames. It is noteworthy that there exists a slight difference between road traffic anomalies and road traffic accidents according to [5], and this paper will not delve into the detailed differences between them, so the word “traffic accidents” in this paper means “traffic anomaly”, which aims to detect time windows of road traffic anomaly containing road traffic accident scenes.

Currently, methods for detecting traffic anomalies from a surveillance perspective can be broadly categorized into two types based on the number of modalities: single visual modality methods and multi-modal representation methods. Single-visual-modal methods rely solely on video data to train models, without providing additional modality data. These models detect anomaly frames in the test set by leveraging visual representation differences between anomaly frames and normal frames present in the training set, similar to universal video anomaly detection. But it is important to note that the detection targets in video anomaly detection [6] are broader than those in road traffic anomaly detection, and thus, it may not adequately capture the unique characteristics of road traffic anomaly. Although video anomaly detection methods can be used for comparative analysis with road traffic anomaly detection methods, they are not suitable for direct application in road traffic anomaly detection.

In recent years, numerous visual-language pre-trained models [7,8,9,10,11] have been proposed, achieving remarkable results in visual tasks by leveraging natural language knowledge, such as image classification [12], object detection [13,14], semantic segmentation [15,16], and image retrieval [17,18], in video domain, such as video recognition [19,20], video retrieval [21,22], and so on. Among these models, the CLIP model [23] stands out as a significant representative, excelling at various visual tasks through semantic alignment between texts and images. For the traffic anomaly detection task, introducing the text modality enables models to better learn key information about traffic anomalies from a large volume of complex visual data [24,25,26,27], thereby enhancing anomaly detection accuracy. However, research on traffic anomaly detection from a text–visual multimodal perspective remains insufficient, as exemplified by article [27], which, based on the CLIP model, aligns visual data with corresponding textual data from a driving perspective to detect road traffic anomalies. Nevertheless, as previously mentioned, there are significant differences between traffic anomaly data from surveillance and driving perspectives. Therefore, it remains necessary to explore and validate whether the CLIP model could enhance its learning capabilities regarding traffic anomaly data from a surveillance perspective by incorporating text information, and whether eye-gaze data from a surveillance perspective could assist the visual-language model in improving its representation capabilities for traffic anomaly detection.

Based on the analysis above, this paper first provides textual annotations of the previously proposed road traffic anomaly dataset from a surveillance perspective, TADS [4]. In detail, to enable the model to better learn the differences between anomaly and normal videos with text assistance, we annotated each video segment in TADS with two types of text descriptions: one for the time windows when the anomaly starts before (normal video portion) and another for the time windows during the anomaly happens (anomaly video portion). Additionally, we constructed two mechanisms for text-assisted learning: coarse-grained and fine-grained. Furthermore, based on the CLIP model, we propose a new method for detecting road traffic anomalies from a surveillance perspective using multimodal visual-language data. Overall, this method encodes video frames and corresponding text using the CLIP model, then performs temporal fusion of the video frame features, and finally calculates the contrastive loss between the video features and their corresponding text features within each group. Lastly, to investigate whether eye-gaze data could be introduced to assist the model’s learning, we compute the cross-attention maps between the last frame (target frame) and the corresponding text in each group, ensuring that these attention maps align with the corresponding eye-gaze maps, thereby incorporating eye-gaze data to aid the model’s learning.

The main contributions of this paper are as follows: Text annotation was performed on the aforementioned surveillance perspective traffic accident detection dataset, TADS. Specifically, two types of text descriptions have been annotated for each video segment in the dataset, covering both the anomaly-before and anomaly portions, to provide textual information that assists model representation learning. Based on the CLIP model and the TADS dataset with text annotations, we have constructed a method for detecting road traffic anomaly from surveillance perspectives. This method incorporates temporal fusion into the original CLIP model, enabling it to be used for representation learning of traffic anomaly videos in road surveillance with a visual-text multimodal manner. Additionally, by introducing a video-text cross-attention mechanism, we explore whether eye-gaze data in the TADS dataset can enhance the representation learning capacity of the model.

The remainder of this paper is organized as follows: Section 2 reviews related work on road traffic anomaly detection; Section 3 outlines the specific steps involved in the text annotation of the TADS dataset; Section 4 describes the specific structure of the detection model proposed in this paper; Section 5 compares the detection performance of our method with other commonly used methods, and validates the effectiveness of each component of our model through the ablation experiments, introduces some discussions about the inference speed of the model; Section 6 concludes the paper and discusses the related research that we can take in the future.

## 2. Related Work

### 2.1. Road Traffic Anomaly Detection Methods

The primary objective of road traffic anomaly detection is to provide real-time alerts for anomalies or accidents occurring in surveillance video. Because of the conceptual similarity between video anomaly detection and road traffic anomaly detection, many video anomaly detection methods have been employed for road traffic anomaly detection.

Common methods applicable for road traffic anomaly detection include supervised classification methods and unsupervised video anomaly detection methods. Supervised classification methods directly categorize anomaly or accident frames and normal or non-accident frames in a video as two distinct classes, training the model using traditional binary classification principles. For instance, Nejdet Dogru et al. [28] used vehicle position and speed data, employing artificial neural networks, support vector machines (SVMs), and random forests to detect accidents. Wei Wang et al. [29] conducted experimental validation of accident detection using partial least squares regression (PLSR) and support vector machines (SVMs) on the I-880 highway dataset in California. Most of these detection methods adopt a relatively straightforward way and simple model structures, comparing only the two categories of accident frames and non-accident frames present in the dataset. Consequently, their representation capabilities are limited, resulting in relatively suboptimal detection performance.

Unsupervised video anomaly detection methods are often implemented using autoencoder models, which are trained to reconstruct normal data in the training set and to identify anomalous data that deviates from the normal data distribution in the testing set. Due to the proximity of traffic anomaly detection to video anomaly detection, this approach can also be applied to road traffic anomaly detection. For example, Liu W. et al. [30] considered the spatiotemporal features of consecutive video frames, employing adversarial training to predict video frames and identifying frames that deviate significantly from the predicted frames as anomalous. This method was verified on several commonly used surveillance video datasets. Chong et al. [31] employed ConvLSTM to extract spatiotemporal features from video data. They trained an autoencoder on normal video segments and subsequently applied it to detect anomalies in videos containing abnormal segments, leveraging the advantages and characteristics of unsupervised detection. These unsupervised video anomaly detection methods have advantages such as rapid processing and lower data annotation requirements (Most of them only require normal video frames for training). However, relying on reconstruction error to determine whether a result is anomalous often leads to a high false-positive rate. Furthermore, this unsupervised training approach struggles to learn the unique characteristics of specific types of anomalies (such as traffic accidents), which seriously affects the detection accuracy of the model in practical applications.

### 2.2. Visual Text Multimodal Learning for Video Anomaly Detection

In recent years, multimodal visual–text learning [7,8,9,10] has achieved significant success across various visual tasks, such as image classification, object detection, semantic segmentation, and image retrieval. Consequently, many researchers have conducted studies on visual tasks using visual-text pre-trained models. Currently, the CLIP model [23] stands out as an important pre-trained model that achieves semantic alignment between visual and textual data by contrasting a vast number of image-text pairs. Numerous researchers have conducted extensive studies on various visual-related tasks based on this CLIP-like model.

M. Wang et al. [32] formulated video action recognition as a video-text matching problem and proposed ActionCLIP, which employs multi-modal learning to enable zero-shot action recognition. H. Luo et al. [33] introduced CLIP4Clip, which transfers knowledge learned from the CLIP model in an end-to-end manner to address video-language retrieval tasks. W. Wu et al. [19] leveraged knowledge from open-source visual-language pre-trained large models to tackle video recognition tasks. These kinds of studies demonstrate that large visual-language pre-trained models, due to their incorporation of essential visual-language associative knowledge, significantly aid downstream visual-related tasks. As a result, some researchers have begun to explore whether these visual-language pre-trained models could also assist in video anomaly detection tasks [24,25,26]. P. Wu et al. [34] proposed VadCLIP, which fully utilizes the fine-grained associations between visual and textual data inherent in the CLIP model, designing a dual-branch detection structure for weakly supervised video anomaly detection. Experimental results on two public datasets indicate that VadCLIP achieves optimal performance in both coarse-grained and fine-grained weakly supervised video anomaly detection. Rongqin Liang et al. [27] developed TTHF based on the CLIP model, focusing on traffic anomaly detection from the driver’s perspective scenarios by assessing the correlation between visual and text features. Although visual-text multimodal pre-trained models are being applied to video anomaly detection tasks, the increasing use of the CLIP model in important downstream visual tasks has not yet led to significant research leveraging the associations between text and visual data for traffic anomaly detection, particularly in the context of surveillance scenarios. It is still necessary to investigate whether visual-language pre-trained models could contribute to road traffic anomaly detection from surveillance perspectives and how to design detection methods that better leverage these models for effective traffic anomaly detection in such contexts.

## 3. Text Annotation on the TADS Dataset

To make better use of the associative knowledge between visual and text data learned from visual-text pre-trained models for distinguishing traffic anomaly frames from normal frames in videos, this paper introduces text annotations for the surveillance perspective traffic anomaly detection dataset previously mentioned, TADS [4]. The text annotation information is provided at two levels of granularity: coarse-grained and fine-grained.

Coarse-Grained Text Annotations: Considering the objective of distinguishing between traffic anomaly frames and normal frames in the video, the traffic anomaly frames, normal frames, and their corresponding text descriptions are aligned within the dataset. Since this kind of text description is intended solely to differentiate between traffic anomalies and normal conditions, and is not specifically related to a particular traffic scene in the video, it is referred to as coarse-grained text annotation. For brevity, refer to article [27]. The coarse-grained text descriptions used in this paper consist of the following two types:

(1) Traffic Anomaly Description: “A traffic anomaly occurred in the scene”. For aligning anomalous video representations with text representations during the training process, all features of traffic anomaly videos are aligned with the text features of this sentence.
(2) Normal Scene Description: “The traffic in this scenario is normal”. For aligning normal video representations with text representations during the training process, all features of videos prior to the occurrence of any traffic anomalies are aligned with the text features of this sentence.

By employing this text setup and alignment approach, the model can focus on differences in video representations between traffic anomalies and normal scenarios, thereby achieving a coarse-grained distinction between traffic anomalies and normality.

**Figure 1:**
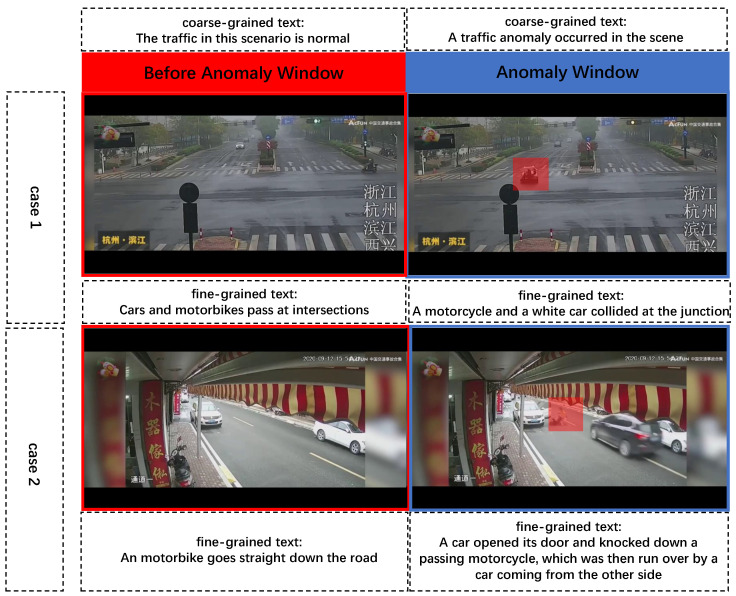
Examples of coarse-grained and fine-grained text annotations. Note: The Chinese texts on the frames represent information related to the surveillance camera or the specific scene.

Fine-Grained Text Annotations: While the aforementioned coarse-grained text annotations facilitate basic differentiation between traffic anomalies and normal traffic scenes, they suffer from the limitation of a single text description (with all video data in the dataset corresponding only to these two sentences). This lack of diversity in text descriptions hinders the model’s ability to learn the rich semantic contexts present in the videos. Therefore, to assist the model in learning the abundant visual information contained within the extensive video data in dataset, and to verify whether this rich visual information aids the model in distinguishing between traffic anomalies and normality with the help of the texts, this paper annotates each segment of the training videos in the dataset with two types of text descriptions: one for the normal scene (the portion of the video prior to the occurrence of the traffic anomaly) and another for the traffic anomaly (the portion of the video from the beginning to the end of the traffic anomaly). Since these text descriptions detailed the specific traffic scene information for each video segment and corresponded with each video segment one-to-one, they are referred to as fine-grained text annotations. It is important to note that fine-grained normal scene descriptions do not include any terms related to traffic anomalies. Examples of coarse-grained and fine-grained text annotations are illustrated in Figure 1.

Following this approach and text-annotation setup, this paper has provided the TADS dataset with a total of 1448 fine-grained text descriptions, comprising 648 traffic normal scene descriptions and 800 traffic anomaly descriptions. It is important to note that the number of normal scene descriptions does not equal the number of traffic anomaly descriptions, as some video segments do not include a portion of the traffic normal scene prior to the occurrence of the traffic anomaly. Additionally, there are two coarse-grained text descriptions, resulting in a total of 1450 text annotations.

**Figure 2:**
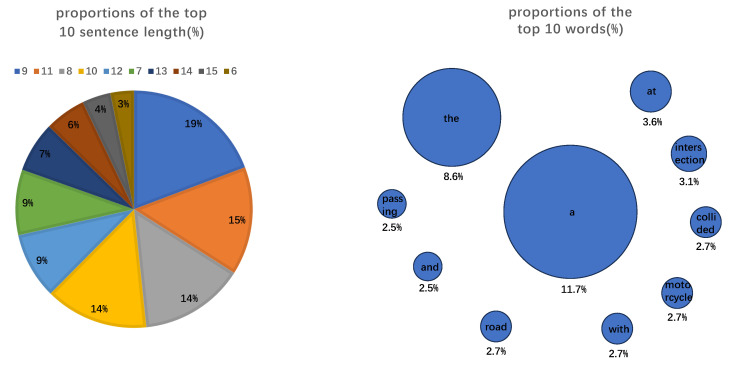
Distribution of lengths of sentences and frequencies of words annotated in the TADS dataset.

Among the 1450 text annotations, the sentence lengths and words used are determined by the real scenes captured in the video segments within the TADS dataset. Based on this, this paper statistically analyzes sentence lengths (represented by the number of words in a sentence) and the distribution of high-frequency words across the 1450 text annotations, as illustrated in Figure 2. Among them, the sentence lengths and high-frequency words were selected from the top 10 with the highest frequency of occurrence for statistical display.

The left part of Figure 2 shows the statistical distribution of annotated text sentence lengths. In the legend, sentence length 9 corresponds to the blue area in the upper right corner of the pie graph, which is 19%, and so on in a clockwise direction. The right part of Figure 2 shows the statistical distribution of word frequencies in the annotated texts; the larger the area of the circle, the higher the frequency of the word.

## 4. The Detection Approach

**Figure 3:**
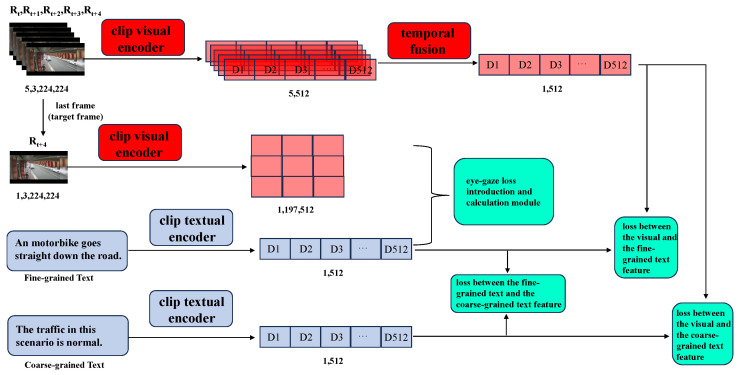
Overview of our proposed detection model. Numbers below each feature in the figure represent the dimension information correspondingly.

This paper extracts videos and their corresponding text features primarily using the visual-language pre-trained model CLIP. Unlike the original CLIP model, which processes a single image, the visual encoding module in this paper handles continuous video frames, so we made some modifications needed to adapt to our specific application compared to the original CLIP. An overview of the model is illustrated in Figure 3.

### 4.1. Multimodal Feature Extraction

Specifically, for a sequence of five consecutive video frames in dataset $F_{t}$, $F_{t+1}$, $F_{t+2}$, $F_{t+3}$, $F_{t+4}$, the visual encoding component of the CLIP model is applied to obtain the corresponding visual features ($F_{t}^{\prime }$, $F_{t+1}^{\prime }$, $F_{t+2}^{\prime }$, $F_{t+3}^{\prime }$, $F_{t+4}^{\prime }$, respectively) for every single frame. Since the video input consists of continuous video frames, the temporal relationships between these frames are crucial. This paper employs a transformer-based fusion approach to temporally integrate the features of consecutive frames in the input video [32], resulting in the final visual feature representation *E*. The ablation experiments presented in Section 5.4 further validate the effectiveness of the temporal fusion module. For the text feature extraction component, the two types of text descriptions (coarse-grained and fine-grained) corresponding to the input video are processed by the text encoding part of CLIP to obtain the coarse-grained text feature *CG*_*t* and the fine-grained text feature *FG*_*t*.

### 4.2. The Introduction of the Eye-Gaze Map

Given the unique visual eye-gaze attention data contained in the TADS dataset, this paper explored whether this visual eye-gaze data could assist in aligning visual and text features, thereby improving the traffic anomaly detection performance of the model.

**Figure 4:**
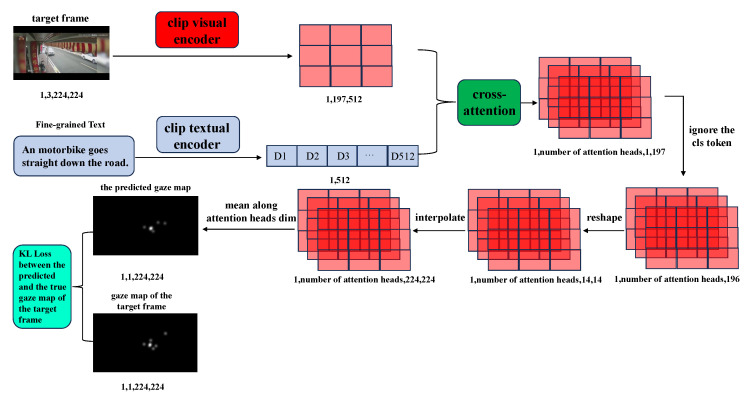
Eye-gaze assisted learning diagram. Numbers below each feature in the figure represent the dimension information correspondingly.

Since visual attention heatmaps represent the spatial focus areas of observers while watching surveillance videos, it is intuitive to expect that, for video frames within the range of traffic anomalies, the attention areas indicated by the heatmaps often overlap with the spatial regions where traffic anomalies or accidents occur. Conversely, for video frames previous to the traffic anomaly interval, i.e., the normal scene interval, the attention areas in the heatmaps are generally not meaningful or unrelated to the traffic anomaly context. To investigate whether visual eye-gaze data can improve detection performance, this paper first defines the last of the five consecutive frames as the “target frame”. Subsequently, the “target frame” and its corresponding fine-grained text are fed into the visual and text encoding modules of the CLIP model to extract the visual feature *V*_*t* and the fine-grained text feature *FG*_*t*, respectively. The obtained visual feature *V*_*t* is then fused with the corresponding fine-grained text feature *FG*_*t* through a cross-attention module. It is important to note that this cross-attention fusion module is not designed to enhance the representation of the visual feature *V*_*t* based on its relationship with the corresponding fine-grained text feature *FG*_*t*; rather, it aims to generate the cross-attention map *G*′_*t* between *V*_*t* and *FG*_*t*. Once the attention map *G*′_*t* corresponding to the “target frame” is obtained, a loss calculation can be performed between the eye-gaze heatmap *G*_*t* corresponding to the “target frame” and the *G*′_*t* aforementioned. This process guides the model to focus on specific regions of traffic anomalies within the video frames during the traffic anomaly interval, thereby exploring the effectiveness of the eye-gaze heatmap in improving the detection performance of the model. The details of the introduction of the eye-gaze heatmap are illustrated in Figure 4.

### 4.3. Loss Function and Traffic Anomaly Discrimination

Our detection method considered the relationships between visual features, text features at different granularities, and output attention maps. Accordingly, we consider four types of loss functions below:

#### 4.3.1. Loss Between Visual and Coarse-Grained Text Features

After deriving the visual features *E* of the input video consisting of five consecutive frames and the corresponding coarse-grained text features *CG*_*t* as described in Section 4.1, we need to align *E* with *CG*_*t*. Since there are only two kinds of coarse-grained texts, in this situation, the same type of coarse-grained text within the same batch may correspond to multiple different video inputs. Therefore, following the approach outlined in the article [32], we employ Kullback-Leibler KL divergence to align the visual features *E* of different input videos within the same batch with their coarse-grained text features *CG*_*t* correspondingly. This loss can be expressed as

(1) $$loss-v2c=\frac{1}{2}E_{(v,c)∼D}\left[KL\left(p^{v2c}\left(v\right),q^{v2c}\left(v\right)\right)+KL\left(p^{c2v}\left(c\right),q^{c2v}\left(c\right)\right)\right]$$

#### 4.3.2. Loss Between Fine-Grained Text and Coarse-Grained Text Features

Since this paper utilizes both coarse-grained and fine-grained texts, we designed a loss function that enables the text feature extraction module to learn the semantic differences between traffic anomalous and traffic normal conditions within a single text modality. This loss function aligns the fine-grained text features with the two kinds of coarse-grained text features.

Similar to the approach described in Section 4.3.1, the same type of coarse-grained text within the same batch may correspond to multiple different fine-grained texts, so we also employ Kullback–Leibler (KL) divergence to align the fine-grained text features *FG*_*t* with their corresponding coarse-grained text features *CG*_*t*. So the calculation about this loss is as follows:

(2) $$loss-f2c=\frac{1}{2}E_{(f,c)∼D}\left[KL\left(p^{f2c}\left(f\right),q^{f2c}\left(f\right)\right)+KL\left(p^{c2f}\left(c\right),q^{c2f}\left(c\right)\right)\right]$$

In Equations (1) and (2), v2c and f2c denote the correspondence between visual and coarse-grained text features, and between fine-grained text and coarse-grained text features, respectively. *D* represents the entire training dataset, while *p* and *q* stand for the similarity values and ground-truth employed in the alignment computation of different features, respectively.

#### 4.3.3. Loss Between Visual and Fine-Grained Text Features

The visual features *E* of five consecutive frames of input video and the corresponding fine-grained text features *FG*_*t* are derived according to the process described in Section 4.1. Subsequently, we consider aligning *E* with *FG*_*t*. This alignment enables the visual and textual feature-extraction modules to better comprehend the semantic relationships between visual and textual factors in traffic scenarios. Unlike the process discussed in Section 4.3.1 and Section 4.3.2, each fine-grained text serves as the only description of the corresponding video input, so in this case, each visual feature *E* of the input videos within the same batch corresponds to its respective fine-grained text feature *FG*_*t* one-to-one. Therefore, referring to the loss used in the original CLIP model [23] where the different inputs are assigned to the different fine-grained texts, we employ the cross-entropy loss function to align the visual features *E* of different input videos within the same batch with their certain fine-grained text features *FG*_*t*. So this loss can be expressed as follows:

(3) $$loss-v2f=\frac{1}{2}[\;\mathrm{cross\_entropy}\left(f@v^{⊤},\;\mathrm{labels}\;\right)+\;\mathrm{cross\_entropy}\left(v@f^{⊤},\;\mathrm{labels}\right)]$$

In Equation (3) above, v2f represents the correspondence between visual and fine-grained text features, and labels represent the ground-truth in the alignment calculation under the one-to-one situation.

#### 4.3.4. Loss Between Attention and Eye-Gaze Maps

Eye-gaze maps are important data components in the TADS dataset. They are obtained by recording the eye focus areas of multiple human observers when they watch the dataset with an eye movement recorder, and then processed through the average Gaussian blurring operation. Unlike the conventional attention mechanism, which treats video frames as spatial annotations, eye-gaze maps represent the focus areas of human observers and provide useful prior knowledge. Details about the eye-gaze maps in the dataset can be found in [4].

To leverage the extensive eye-gaze data in the TADS dataset, this paper investigates whether regions of these eye-gaze maps can influence the fusion of visual and textual features, thereby enhancing the detection performance of the model. As mentioned in Section 4.2, we perform cross-attention fusion between the visual features *V*_*t* of the target frame and the corresponding fine-grained text features *FG*_*t*. The resulting multi-head attention maps are then converted to the final fused attention map *G*′_*t* by interpolation, scaling, and averaging across the multi-head dimension operations. Finally, referring to the research and practices in driving attention prediction [35], we employ Kullback–Leibler (KL) divergence loss to approximate the fused attention map *G*′_*t* with the ground truth eye-gaze map *G*_*t* of the target frame correspondingly. This can be expressed as follows:

(4) $$loss-gaze\left(\mathrm{G\_t},\mathrm{G\prime \_t}\right)=\sum_{i}\mathrm{G\_t}\left(i\right)\log\left(\varepsilon +\frac{\mathrm{G\_t}(i)}{\varepsilon +\mathrm{G\prime \_t}\left(i\right)}\right)$$

Unlike the usage in [35], in order to simplify the calculation, we only use the KL divergence, which is used to bring two distributions closer, to approximate the two kinds of maps. So in Equation (4), *G*_*t* and *G*′_*t* represent the fused attention map and the ground truth, respectively. The summation index i spans across image pixels, and ϵ is a small constant that ensures numerical stability.

Based on the formulas and description above, we included pseudocode of the core of the implementation of our method in Algorithm 1.

In Algorithm 1, the input_data consists of four types of data as required mentioned above, namely consecutive t frames of video frame group, the eye-gaze maps, fine-grained texts, and coarse-grained texts corresponding to the target frames. CLIP_visual, CLIP_text, temporal_fusion, and cross_ttn_i2t in the pseudocode represent the visual and text encoding modules of the CLIP model in this paper, the temporal fusion module for temporal representation of frame groups, and the cross attention module for fusing visual and text features, respectively. We get and reshape the attention maps by the attn_transfer function. Loss-v2c, loss-v2f, loss-f2c, and KL-loss are the four loss functions described in Section 4.3, which are weighted and summed to obtain the training loss.
**Algorithm 1** The pseudocode of training process**Require:**  frames_group; **Require:**  gaze_map; **Require:**  fine_grained_texts; **Require:**  coarse_grained_texts;
 **for** each input_data in dataLoader **do**
     frame_target = frames_group[:, -1, :, :, :]
     frame_emb_f, _ = CLIP_visual(frames_group)
     frame_emb = temporal_fusion(frame_emb_f)
     _, frame_emb_withpn = CLIP_visual(frame_target)
     coarse_emb = CLIP_text(coarse_grained_texts)
     fine_emb = CLIP_text(fine_grained_texts)
     fine_grained_out, the_atten = cross_attn_i2t(frame_emb_withpn, fine_emb)
     atten_calcul = attn_transfer(the_atten)
     loss_coarse = loss-v2c(frame_emb, coarse_emb)
     loss_fine = loss-v2f((frame_emb, fine_emb))
     loss_coarse2fine = loss-f2c(fine_emb, coarse_emb)
     loss_gaze = KL-loss(gaze_map, atten_calcul)
     loss = weighted_sum(loss_coarse, loss_fine, loss_coarse2fine, loss_gaze)
     loss.train()
 **end for**
▷ (b,t,c,h,w)
▷ (b,1,h,w)
▷ (b,77)
▷ (b,77)

▷ take the last frame as the target


#### 4.3.5. Traffic Anomaly Score and Discrimination

During the inference/testing phase, there are no fine-grained text descriptions or eye-gaze maps of the target frames in the input video. Therefore, as described in Section 4.1, we extract features from the input video and perform temporal fusion to obtain the visual features *E*. Then, we extract text features from two coarse-grained sentences which represent traffic anomaly and traffic normality respectively: “A traffic anomaly occurred in the scene.” and “The traffic in this scenario is normal." The two coarse-grained sentences result in a text feature pair (a_t,n_t). We normalize the features *E* and the text feature pair (a_t,n_t), and then calculate their cosine similarities as (sim_a,sim_n) respectively. Finally, the cosine similarities (sim_a,sim_n) are converted to the pair (s_a,s_n) by the softmax operation. Here, s_a is regarded as the traffic anomaly score of the target frame in the input video. Target frames with traffic anomaly scores greater than 0.5 are classified as traffic anomalous frames. The process can be denoted as:

(5) $$\left(\mathrm{s\_a},\mathrm{s\_n}\right)=F.\;\mathrm{softmax}\;\left(\;\mathrm{similarity}\;\left(E,\mathrm{a\_t}\right)\;,\;\mathrm{similarity}\;\left(E,\mathrm{n\_t}\right)\right)$$

## 5. Experiments and Analysis

### 5.1. Evaluation Metrics

This paper employs the Area Under the Curve (AUC) as the main evaluation metric. AUC represents the area under the Receiver Operating Characteristic (ROC) curve, which is calculated using the confusion matrix across all thresholds to obtain the True Positive Rate TPR and False Positive Rate FPR. A larger area under the ROC curve indicates a higher AUC for the model, signifying better performance. Furthermore, when there are multiple video segments in the test set, we refer to [36] to adopt two types of AUC metrics: The AUC calculated from concatenating all frames in the test set is referred as “micro AUC,” while the AUC values computed for each video segment, followed by averaging across all segments, are termed as “macro AUC.” Using both “micro AUC” and “macro AUC” can provide a more comprehensive evaluation of the detection performance of the model.

In addition to AUC, this paper uses accuracy as an evaluation metric. The accuracy value is computed as the proportion of traffic anomaly video frames that are correctly classified by the model. The decision threshold is set at 0.5 in our experiments, so we classify frames with traffic anomaly scores greater than 0.5 as traffic anomaly frames. So the detection accuracy is defined as the ratio of correctly detected frames to the total number of frames in the test set, which can be written as follows:

(6) $$accuracy=\frac{number\;of\;correctly\;detected\;frames}{total\;number\;of\;frames\;in\;the\;testing\;set}$$

### 5.2. Experimental Setup

All experiments conducted in this paper were based on the Ubuntu 20.04 operating system, implemented based on the PyTorch 1.10 framework, with an Nvidia 3090 GPU. A sequence of five consecutive frames of the same category are combined into an input video frame group, which means the target frame (the last frame of the video frame group) and the preceding frames either both belonging to the traffic normality or both belonging to the traffic anomaly. Both the input video frames and the eye-gaze maps were resized to 224 × 224 pixels, with a batch size of 8. The AdamW optimizer was employed alongside the WarmupCosineAnnealingLR learning rate adjustment strategy. Since this paper extracts the initial visual-textual features from the input data based on the CLIP model, a smaller initial learning rate of $5\times 10^{-5}$ was set for the CLIP-based components of the model. In contrast, the initial learning rate for the remaining parts of the model was set higher, at $1\times 10^{-3}$. The total number of training epochs was set to 10. The four loss functions described in Section 4.3 were assigned weights of 1.0, 0.3, 0.15, and 0.8, respectively. It is worth noting that this paper constructed a special data structure consisting of the video frame group, eye-gaze map, coarse- and fine-grained texts correspondingly, to accelerate the process of data fetching by dataLoader, so as to speed up the process of training.

### 5.3. Performance Comparison and Analysis

#### 5.3.1. Competitors

Based on the discussions in Section 2, several models from both supervised learning classification methods and unsupervised video anomaly detection methods can be selected for performance comparison. This paper chooses two representative methods for supervised learning classification: ResNet50, which extracts spatial features from video frames using the ResNet50 [37] architecture and subsequently employs a fully connected layer for binary classification; and LS-ResNet50, which utilizes spatial features extracted by ResNet50 and employs an LSTM network for temporal feature extraction, followed by a fully connected layer for binary classification. For the unsupervised video anomaly detection or traffic anomaly detection methods, we select three representative approaches: MNAD [38], which improves the performance of the model by introducing a memory mechanism based on an autoencoder, with two detection modes: reconstruction-based MNAD (recon) and prediction-based MNAD (pred); OLP-TAD [30], which employs a U-Net architecture for frame prediction while considering optical flow information in the temporal dimension, trained using a Generative Adversarial Network (GAN) fashion; and RF-RG [4], which first captures important objects in video frames using object detection, such as vehicles and pedestrians, and then predicts frames based on the captured objects while considering optical flow information as a temporal characteristic and simultaneously predicting the eye-gaze maps of the objects during training, and some experiment results in this paper are referred to the article [4].

#### 5.3.2. Overall Performance Comparison

After training, the detection metrics and performance comparison of each model on the test set of the TADS dataset are presented in Table 1.

The results of various models presented in Table 1 indicate that the method proposed in this paper significantly enhances the detection performance of traffic anomalies from a surveillance perspective. Compared to the previous best method, RF-RG, on the TADS dataset, our approach improves the micro AUC metric by approximately 6%, the macro AUC metric by about 7%, and the accuracy metric by around 4%. We consider that this significant improvement can be attributed to several factors below:

Foundation of visual-text pretrained model: The CLIP model is a large visual-text model trained on a vast number of image-text pairs, demonstrating a powerful ability to extract features that capture the semantic relationships between visual and textual factors. Its effectiveness has been validated in various visual downstream tasks. By fine-tuning the visual and textual feature extraction components of the CLIP model on the TADS dataset, we can extract semantic features that relate traffic scene videos to fine-grained and coarse-grained text descriptions. This semantic relationship is intuitively very helpful for the model in distinguishing between traffic anomaly videos and traffic normal videos.

The introduction of multiple text granularities: Intuitively, traffic scene videos contain more complex semantic information than single images. To effectively differentiate between traffic anomaly frames and traffic normal frames, relying solely on coarse-grained text may lead the model to overlook the rich semantic information related to traffic anomalies present in the input video frames. This information may be crucial to making it easy for the model to detect traffic anomalies. Conversely, if only the fine-grained text associated with the input video is introduced, the model may deviate from its objective of distinguishing between traffic anomalies and traffic normality, becoming entangled in the complex task of aligning videos with fine-grained text, which could negatively affect the model’s detection performance. The introduction of multiple text granularities allows the fine-grained text information corresponding to each video to complement the two coarse-grained text types relevant to the detection task, thereby ultimately enhancing the model’s traffic anomaly detection performance.

Assistance from the eye-gaze maps: As discussed in Section 1, the areas where traffic anomalies or accidents occur often occupy a smaller spatial region within the entire frame from the surveillance perspective. Therefore, effectively utilizing eye-gaze maps (observer attention maps) could help the model focus on important areas within the frame, reducing the uselessness and redundancy of background information. The approach of employing object detection first, followed by anomaly detection in previous studies [39], may have demonstrated this principle. In our experiments, we did not first perform object detection on the input video frames, as in prior methods; however, we still achieved favorable detection results, likely due to the use of eye-gaze maps from the TADS dataset. These eye-gaze maps often align closely with the traffic anomaly text on the semantic point during incidents, and our approach also relies on the semantic correlation between visual and textual factors to detect traffic anomalies. Thus, the use of eye-gaze maps as auxiliary information may further enhance the detection performance of the model.

#### 5.3.3. Performance Comparison on Various Categories of Traffic Anomaly

**Figure 5:**
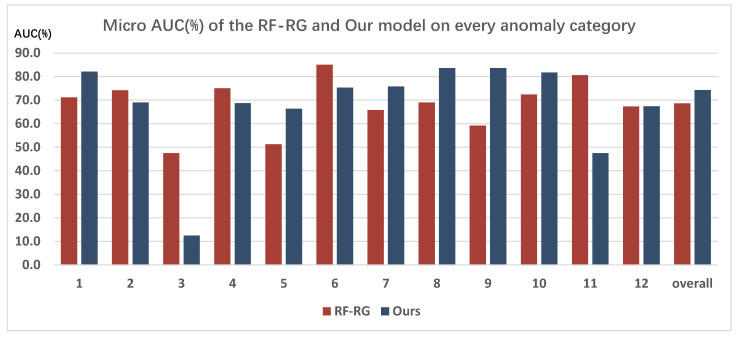
Performance comparison on various categories of traffic anomaly.

To comprehensively evaluate the performance of the proposed model, this paper compares it across various categories of traffic anomaly, and selects the second-best model, RF-RG, from Table 1 for comparative analysis. It is important to note that the TADS dataset categorizes traffic anomaly into 12 different types based on the anomalous objects involved. For instance, category 1 refers to “collisions between small vehicles and road infrastructure,” while category 2 pertains to “collisions between large vehicles and road infrastructure,” among others. Detailed definitions of each category can be found in [4]. The comparative results of the detection for each category of traffic anomaly are illustrated in Figure 5, with the evaluation metric being Micro AUC(%).

As shown in Figure 5, the model proposed in this paper demonstrates strong performance across the majority of anomalous categories present in the TADS dataset. Among the 12 different types of anomaly, performance improvements were observed in 7 categories (anomalous categories 1, 5, 7, 8, 9, 10, and 12), with varying degrees of enhancement. For instance, anomalous category 1 improved by 9% and category 5 by 15%, resulting in an overall improvement of approximately 6%.

In fact, different anomalous categories often exhibit distinct motion characteristics. Improving the detection performance of the model by focusing solely on visual data may lead to high metrics results for specific anomaly categories, while yielding unsatisfactory performance for others. Employing a multimodal visual–text learning approach can help the model capture common semantic features of traffic anomalies from both visual and textual perspectives, thereby improving the detection ability across multiple anomalous categories.

However, it is also shown that the model proposed in this paper achieved lower detection performance for certain anomalous categories, such as categories 2, 3, 4, 6, and 11. We guess this may be attributed not only to the inherent challenges of detecting traffic anomalies in the surveillance scenario or within the TADS dataset but also to the limited quantity and proportion of those specific anomalous categories in the dataset. For example, the proportions of anomalous categories 2, 3, and 11 in the training set are only 2%, 0.25%, and 2%, respectively. Such a low quantity ratio could not be conducive to the visual-text multimodal learning way, which is more complex and usually needs more training data to work, ultimately affecting the detection performance of the model on these categories.

In summary, the detection metrics for most anomalous categories indicate that the visual-text multimodal learning approach proposed in this paper achieves better detection performance. This also demonstrates the feasibility of incorporating textual information and knowledge for road traffic anomaly detection from a surveillance perspective.

#### 5.3.4. Qualitative Results

**Figure 6:**
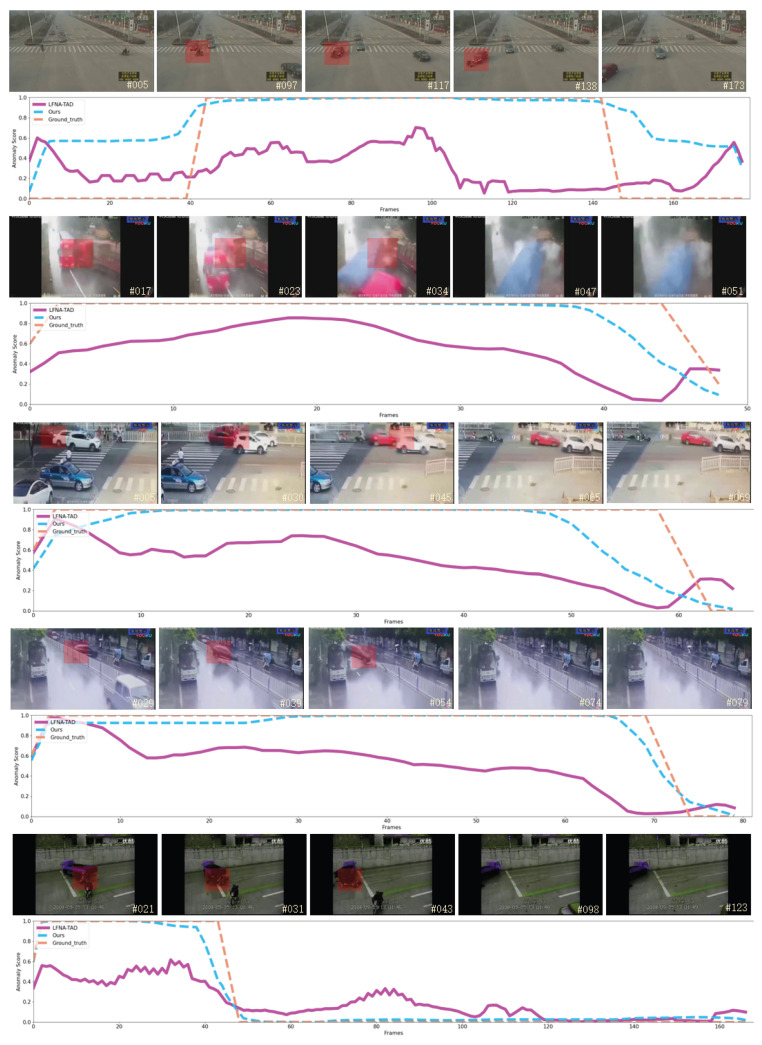
The visualization of anomaly score curves for traffic anomaly detection of these two models on the TADS dataset. Note: The non-English terms on the frames represent information related to the surveillance camera or the TADS dataset itself, refer to [4] for more details.

This paper selects the second-best model, RF-RG, from Table 1 for qualitative comparison to further illustrate the detection capability of our model. Five video segments from the test set are chosen as qualitative examples. The results are presented as anomaly score curves for the models on these examples, alongside specific sample frames, as shown in Figure 6.

In Figure 6, the frames corresponding to traffic anomaly intervals are marked with a red box to indicate the anomaly spatial regions, and we refer to the test samples in Figure 6 as test samples 1, 2, 3, 4, and 5, respectively, from top to bottom. The following points can be observed from the qualitative comparison in Figure 6: The RF-RG model, identified as the second-best method in Table 1, achieves a commendable detection AUC value of 68.6%. However, the RF-RG anomaly score curves on these test samples are not optimal, indicating that this method has not fully captured the distinguishing features between traffic anomaly and normal videos. The proposed method in this paper not only attains a high detection AUC value but also produces anomaly score curves that closely align with the ground truth labels. In many instances, the trends of curves of our method are also consistent with the ground truth, suggesting that the proposed method effectively learns discriminative features. However, it is noteworthy that in certain intervals of the test samples, the anomaly score curves produced by the proposed method exhibit significant discrepancies from the ground-truth labels. For instance, in the normal interval preceding the traffic anomaly in test sample 1, the proposed method also outputs a high anomaly score. Similarly, in test samples 2 and 3, when transitioning from the traffic anomaly interval to the normal interval, the proposed method does not immediately yield a lower anomaly score. We guess that there are two possible reasons for these results. First, detecting traffic anomalies from a surveillance perspective is inherently challenging. As mentioned in the introduction Section 1, traffic anomalies often occupy a small spatial area in surveillance video frames, which poses significant challenges for model training. The second reason would be the unique semantics associated with traffic anomaly. As a specific type of video anomaly, traffic anomalies usually exhibit distinct spatiotemporal characteristics compared to other general video anomalies. Moreover, the beginning and end of traffic anomalies are difficult to define accurately in terms of their semantic implications, making it challenging for the model to perfectly align with the ground truth labels during the intervals near the occurrence and cessation of the traffic anomaly.

### 5.4. Ablation Experiment

#### 5.4.1. Overall Performance for Ablation

To investigate the impact of various components of the proposed model on detection results, this paper sequentially removes the eye-gaze maps, fine-grained texts, and the temporal fusion module successively, and observes the changes in the detection performance. The detailed descriptions of the model variants are as follows:

base-t-f: This variant is based on the final version of the proposed model but excludes the training input of eye-gaze maps. In this case, the training loss no longer includes the eye-gaze loss and is instead composed of a weighted sum of three losses: the loss between visual features and coarse-grained text features, the loss between visual features and fine-grained text features, and the loss between fine-grained text features and coarse-grained text features. The weights among the losses remain unchanged.

base-t: Building on the base-t-f model, this version further removes the training input of the fine-grained texts. Consequently, the training loss will only include the loss between visual features and coarse-grained text features. Specifically, the visual features are extracted from the continuous five frames of video using the CLIP model, and after temporal fusion, the fused visual features are coupled with the corresponding coarse-grained text features to compute the loss.

base: This model is derived from the base-t model by removing the temporal fusion module. In this case, for the input of the continuous five frames, only the last frame is sent to the visual encoding module of the CLIP model, and the resulting features are directly coupled with the corresponding coarse-grained text features to compute the loss.

It is important to note that, in this ablation, the final model is referred to as base-t-f-g. Since none of the ablation models include fine-grained text or eye-gaze maps during testing, the processes are consistent across the base-t, base-t-f, and base-t-f-g models. Specifically, all models input the five consecutive frames into the visual encoding part of the CLIP model, followed by feature fusion through the temporal feature fusion module, with the resulting features serving as the visual features for the target frame. These features are then used to calculate similarity with the coarse-grained text features to derive the anomaly score for the target frame. The base model, lacking the temporal feature fusion component, directly feeds the last of the five consecutive frames into the visual encoding part of the CLIP model to obtain visual features for the target frame, which are then coupled with coarse-grained text features to compute the anomaly score.

The detection results for each ablation model are presented in Table 2.

The results presented in Table 2 indicate that the final model, base-t-f-g, achieved excellent performance across all three evaluation metrics. Although the accuracy metric for base-t-f-g is not the highest, it is also very close to the best: base-t-f. In the field of anomaly detection, the AUC metric is more commonly used than the accuracy metric, with micro AUC slightly more common than macro AUC. Therefore, according to the micro-AUC results, the temporal fusion component, fine-grained texts, and eye-gaze maps are indeed beneficial.

#### 5.4.2. Performance on Various Categories of Traffic Anomaly for Ablation

**Figure 7:**
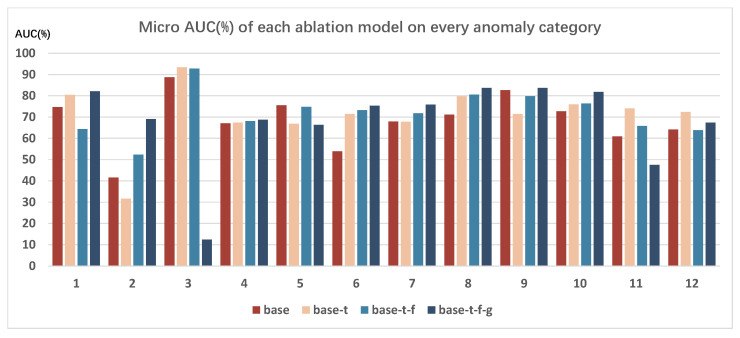
Ablation performance comparison on various categories of traffic anomaly.

To conduct a more comprehensive ablation comparison of the model variants, similar to Section 5.3.3, this paper performed an ablation analysis of the detection results for various categories of traffic anomalies on the TADS test set. The results are presented in Figure 7. It is worth noting that among the three evaluation metrics used in this paper, micro AUC is the most commonly used in the field of anomaly detection, so all results shown in Figure 7 are reported as micro AUC values.

Based on the detection results across various traffic anomaly categories, the base-t-f-g model achieved the best performance for the majority of them, including categories 1, 2, 4, 6, 7, 8, 9, and 10. For the categories with less satisfactory detection results, such as categories 3, 5, 11, and 12, we think the reasons are multifaceted. As analyzed in Section 5.3.3, in addition to the inherent challenges of detecting traffic anomaly from a surveillance perspective, the limited amount of data for certain anomaly categories in the TADS dataset could also be an important reason. We think this visual-text multimodal learning approach may struggle to effectively learn the desired visual-text features when the data volume is insufficient. Overall, the ablation results across various categories of traffic anomalies indicate that the temporal fusion component, fine-grained text, and eye-gaze maps are beneficial for detection performance.

### 5.5. Zero-Shot and Inference Speed

#### 5.5.1. Zero-Shot and Fine-Tuning

As a popular visual-language pretrained model, CLIP has been trained with a large number of image-text pairs, which enables it to perform well in general visual tasks such as image classification, even in a zero-shot manner. To better understand the research problem of this paper, experiments were conducted on the TADS test set using the CLIP model in a zero-shot manner in this section. The visual and text encoding modules of the CLIP model were consistent with the variants used in the experiments of Section 5.3.2. The comparison of results is shown in Table 3.

From the results in Table 3, it can be seen that the results of the CLIP model with zero_shot on the TADS test set are not satisfactory enough, compared to the performance of the model we have trained, and its two kinds of AUC results are both low, even lower than the performance of the base variant model in the ablation experiment section in Section 5.4.1. We consider that this may be attributed to the challenges of the problem of road traffic anomaly detection from a surveillance perspective in the TADS dataset. By comparison, the detection performance of the model after training has improved significantly, indicating the necessity of training on the downstream task when introducing the CLIP model into our problem.

#### 5.5.2. Inference Speed

The processing and response rate under the real usage environments are also very important for applications from the surveillance perspective, especially for the detection of road traffic anomalies. Therefore, the processing speed of the model in the testing stage is statistically measured in this section, with processing and response speeds reported in FPS (Frames Per Second), as shown in Table 3.

As shown in Table 3 about the inference speed, the FPS value of our model during testing is not very high, but the value of 54 could meet the requirements of real-time processing roughly. However, it is worth noting that the frame processing during testing in the paper is performed offline; the frame processing speed (FPS) is generally further reduced in online processing mode under real-world application environments. We attributed the lower FPS performance of our model to the increased complexity introduced by the additional modules—particularly the temporal fusion module and the eye-gaze loss calculation module—compared to the original CLIP model in a zero-shot setting. It is obvious that the transformer-based model is usually inherently heavy, so the transformer-based temporal fusion module and the cross-attention module integrated into the eye-gaze loss calculation in this work significantly increase the model’s final complexity, thereby increasing the running time and negatively impacting the model’s real-time performance. Accordingly, future work could be focused on the lightweight and real-time performance of the model. For example, attempting to replace the transformer-based temporal fusion with a convolutional neural network (CNN)-backboned alternative; or redesigning a fully lightweight architecture for the detection task in this paper, reducing the number of sub-modules, and training such a lightweight model via knowledge distillation, with guidance from the output of the current model. These measures are expected to enhance the real-time inference capability of the overall pipeline, making it more suitable for deployment and implementation in practical application scenarios. From the results in Table 3, although the FPS value of the CLIP model when performed in a zero_shot manner has increased compared to our model, its detection performance has also decreased significantly, reflecting a trade-off between the two metrics.

## 6. Conclusions

This paper explored the feasibility of using visual-text multimodal learning to detect traffic anomalies from a surveillance perspective. Specifically, we first performed fine-grained text annotation on the TADS dataset, which is designed for traffic anomaly detection from a surveillance perspective. This involved adding textual descriptions for normal intervals (prior to the occurrence of an anomaly) and for intervals during traffic anomalies occurring for each video sample. Subsequently, we utilized the CLIP model to extract visual features and text features. By employing both coarse-grained and fine-grained texts, we constructed four loss functions to better align visual features with their corresponding text features. Additionally, we trained a cross-attention module between the target frames during the anomaly intervals and their corresponding fine-grained texts, aiming to bring their interrelations closer to the eye-gaze maps associated with the target frames, leveraging the available eye-gaze data in the TADS dataset to assist model learning. The experimental results demonstrate the effectiveness of the proposed model.

However, based on the experimental results, there are at least two avenues for further enhancing the detection capability of the model. Firstly, from the overall detection results, the model achieved a detection AUC value of 74.3%, indicating significant room for improvement compared to results in most general surveillance video anomaly-detection problems. We think it is due to the diverse and complex scenarios present in traffic surveillance videos. Secondly, for certain specific categories of traffic anomalies, the results from the ablation experiments did not fully match expectations. We attribute this to the limitation of the learning capacity of the model and the insufficient amount of data for the corresponding anomaly categories. Therefore, future efforts could focus on enhancing the learning capability of the model and increasing the data volume for certain traffic anomaly categories to further improve the detection performance.

## Figures and Tables

**Table 1:** Overall performance of various models on the TADS testing set. Numbers in bold indicate the best results.

Models	Evaluation Metrics (%)		
	Micro AUC	Macro AUC	Accuracy
Resnet50 [37]	52.7	50.3	55.4
LS-Resnet50	59.0	47.2	61.8
MNAD(recon) [38]	54.1	58.0	53.0
MNAD(pred) [38]	66.8	66.6	63.7
OLP-TAD [30]	61.3	63.7	63.2
RF-RG [4]	68.6	69.1	62.8
Ours	**74.3**	**76.0**	**66.4**

**Table 2:** Overall performance comparison of each ablation model. Numbers in bold indicate the best results.

Models	Evaluation Metrics (%)		
	Micro AUC	Macro AUC	Accuracy
base	69.3	67.8	62.3
base-t	71.0	73.0	64.6
base-t-f	72.4	71.4	**67.2**
base-t-f-g	**74.3**	**76.0**	66.4

**Table 3:** Performance and inference speed comparison of CLIP(zero_shot) and ours.

Models	Evaluation Metrics (%)			Inference Speed (FPS)
	Micro AUC	Macro AUC	Accuracy	
CLIP(zero_shot)	38.6	39.6	39.4	83
Ours	74.3	76.0	66.4	54

## Data Availability

The data that support the findings of this study are available from the author Yachuang Chai upon reasonable request.
